# 

**Published:** 1856-03

**Authors:** 


PUBLISHED MONTHLY, AT $3 PER ANNUM IN ADVANCE.

### ATLANTA MEDICAL AND SURGICAL JOURNAL.

EDITED BY

JOSEPH P. LOGAN, M. D.,
PROFESSOR OF PHYSIOLOGY AND GENERAL PATHOLOGY,

AND

W. F. WESTMORELAND, M. D.,
PROFESSOR OF THE PRINCIPLES AND PRACTICE OF SURGERY.

*Pax et scientia, sed veritas sine timore.*

12642

VOL. I. MARCH, 1856. NO. VII.

ATLANTA, GEORGIA:

PRINTED BY C. R. HANLEITER AND CO.

1856.

☞ Each Number contains sixty-four pages. POSTAGE.—Under 500 miles, 15 cents a year; over 500 miles, 30 cents a year—to be pre-paid quarterly. If not pre-paid, double the above rates.

